# Apical Function in Neocortical Pyramidal Cells: A Common Pathway by Which General Anesthetics Can Affect Mental State

**DOI:** 10.3389/fncir.2018.00050

**Published:** 2018-07-02

**Authors:** William A. Phillips, Talis Bachmann, Johan F. Storm

**Affiliations:** ^1^Faculty of Natural Sciences, Psychology, University of Stirling, Stirling, United Kingdom; ^2^Department of Penal Law, University of Tartu, Tartu, Estonia; ^3^IBMS Department of Physiology, University of Oslo, Oslo, Norway

**Keywords:** general anesthesia, neocortical pyramidal cells, apical amplification, neural correlates of consciousness, noradrenergic arousal, thalamus

## Abstract

It has been argued that general anesthetics suppress the level of consciousness, or the contents of consciousness, or both. The distinction between level and content is important because, in addition to clarifying the mechanisms of anesthesia, it may help clarify the neural bases of consciousness. We assess these arguments in the light of evidence that both the level and the content of consciousness depend upon the contribution of apical input to the information processing capabilities of neocortical pyramidal cells which selectively amplify relevant signals. We summarize research suggesting that what neocortical pyramidal cells transmit information about can be distinguished from levels of arousal controlled by sub-cortical nuclei and from levels of prioritization specified by interactions within the thalamocortical system. Put simply, on the basis of the observations reviewed, we hypothesize that when conscious we have particular, directly experienced, percepts, thoughts, feelings and intentions, and that general anesthetics affect consciousness by interfering with the subcellular processes by which particular activities are selectively amplified when relevant to the current context.

## Introduction: The Issues and Our Hypotheses

General anesthetics are designed to produce a temporary, reversible and harmless loss of consciousness. Advances in our understanding of the ways in which general anesthetics affect mental state may therefore cast light on the neural correlates of consciousness (NCC). Stating the central issue addressed here in this way implies that mental states can be adequately grouped into those that are conscious and those that are not. Though our presentation will proceed on that simplifying assumption, the final section notes that there may be reasons to question it. Furthermore, as consideration of these issues requires the discussion of aspects of conscious experience and neurobiological activity that are closely related, we take care to keep them conceptually distinct. We assume that different aspects of consciousness have different neuronal bases, and that one distinction relevant to both the phenomenology and the neurobiology is that between content and level. Subjective aspects of conscious phenomenology include not only the categorical or semantic content that are related to the content-specific NCC in terms of Koch et al. (2016), but also those aspects that characterize the level of consciousness (Bachmann, 2012). Koch et al. (2016) subsume neuromodulatory and other level-regulating factors under the background conditions for being conscious, which are assumed to enable consciousness without contributing directly to its semantic content. As Bachmann and Hudetz (2014) propose, we assume that these and other modulatory mechanisms affect aspects of subjective experience that are measurable psychophysically by reference to attributes such as clarity, salience, vividness and confidence.

We make no attempt to review the many theories of general anesthesia, but do relate our hypotheses to those of Mashour and Hudetz (2017). These leading researchers distinguish three classes of general anesthetic. One class, such as dexmedetomidine, reduces the “level” of consciousness by effects on “bottom-up” subcortical mechanisms that regulate arousal. Another class, such as ketamine, reduces or distorts the “contents” of consciousness by effects on “top-down” mechanisms that play a major role in specifying semantic content. The effects of ketamine are thought to be particularly revealing because they can be seen as primarily reflecting effects on the contents rather than on the level of consciousness. The third class, such as propofol, is thought to affect both level and semantic content, thus having a dual action that makes them especially useful.

We agree with Mashour and Hudetz (2017) that: (i) it is useful, as a first approximation, to assume that conscious states have both “level” and “content”, as suggested previously by others (e.g., Laureys et al., 1999, 2004); (ii) general anesthetics may affect either or both; and (iii) this casts light on the neural bases of consciousness. They argue that the effects of anesthetics on level and content may be “intertwined” but do not suggest how. We argue that they are intertwined partly because both involve modifications of apical function within the neocortical pyramidal cells whose activity is central to the contents of consciousness and which are also major targets of signals that regulate levels of arousal and consciousness. The existence of a long apical dendrite linking the soma to an apical tuft in layers 1 and 2 of the cortex has been a characteristic of the large neocortical pyramidal cells since their discovery more than a 100 years ago. One consequence of this is that tuft inputs are electrotonically distant from the soma. If the apical dendrite were a passive cable then inputs to tuft synapses would have little or no effect on the generation of action potentials. The apical dendrite is studded with voltage-dependent channels that actively propagate and modify dendritic signals, however, and this can partly compensate for the distance of tuft synapses from the soma, thus approximating a “dendritic democracy” (e.g., Magee, 1999). Recent discoveries (e.g., Sherman, 2012; Larkum, 2013), outlined in more detail below, suggest that our understanding of these active dendritic mechanisms needs to be supplemented, with fundamental implications for our understanding of the neural bases of perception, consciousness, and the mechanisms of anesthesia. Thus, our article aims to contribute to the development of the comprehensive systems-neuroscience approach to anesthesia called for by Mashour and Hudetz (2017).

In brief, the hypotheses outlined below build upon evidence that within many neocortical pyramidal cells, such as thick-tufted layer 5 cells, there is an apical integration zone (AIZ) near the top of their apical dendrite with effects that clearly distinguish the information processing functions of apical from basal inputs. We interpret the available evidence as indicating that a common function of tuft inputs to the AIZ is to amplify or attenuate responses of pyramidal cells to their basal inputs (e.g., Larkum, 2013; Phillips et al., 2016; Phillips, 2017). Thus, in this mode of operation, the selective feedforward receptive fields about which these pyramidal cells reliably transmit information is determined by their basal inputs. Though there are species differences, a preserved feature across mammals is that these feedforward connections avoid layer 1 and terminate most densely in layers 3 and 4, whereas feedback projections are dense in layer 1 (see review by D’Souza and Burkhalter, 2017). We will cite evidence that inputs to tuft synapses in layer 1 come from diverse sources and amplify responses to basal inputs when those responses are relevant to the current activity elsewhere in the system, as signaled by apical inputs. In relation to the distinction between level and content discussed by Mashour and Hudetz (2017), the hypotheses we focus on here imply a distinction between the general level of neocortical arousal and levels of prioritization or salience of particular semantic contents. On this view, the general level of arousal is regulated by subcortical systems, whereas prioritization depends upon locally specific interactions within the thalamocortical system. Our hypotheses are closely related to that of Cauller and Connors (1992) who argue that general anesthesia is a state in which the influence of backward projections to the most superficial cortical layers is suppressed. In support of that view, they cite evidence that a component of the somatosensory-evoked cortical potential that is generated by excitation of layers I and II in awake monkeys and related to their behavior is selectively abolished during unconscious states of slow-wave sleep (Cauller and Kulics, 1988) and general anesthesia (Arezzo et al., 1981). Our hypotheses support and extend the hypothesis of Cauller and Connors by relating it to much recent evidence on the function of input to synapses of the apical tuft and its role in regulating the state of consciousness. “Apical Amplification (AA): Selective Amplification of Pyramidal Cell Outputs by Inputs to Their Apical Dendrites” section reviews evidence that neocortical pyramidal neurons can function in a mode in which contextual information received via the apical dendrites amplifies their action potential output when relevant in the current context. “Evidence and Arguments Relating Apical Function to Conscious State” section summarizes several grounds for associating this selective amplification with conscious state. “Evidence That General Anesthetics Interfere With Apical Function” section reviews evidence indicating that all or most general anesthetics operate by interfering with apical function. Issues that arise from these hypotheses, including their difficulties and inadequacies, are discussed in the final sections.

## Apical Amplification: Selective Amplification of Pyramidal Cell Outputs by Inputs to Their Apical Dendrites

**Figure 1:**
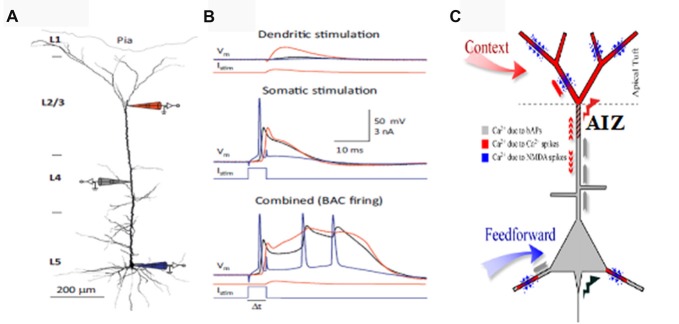
Apical amplification (AA). Evidence from multi-site patch-clamping, and the intracellular processes inferred from such evidence. **(A)** A layer 5 (L5) neocortical pyramidal cell with simultaneous patch-clamp recording in the soma (blue), near the middle of the apical dendrite (gray), and near the top of the apical dendrite. **(B)** Post-synaptic potentials recorded at the three sites to current injected at the apical integration zone (AIZ) (top), in the soma (middle), or at both (bottom). The key observation is that AIZ stimulation, which by itself has little or no effect on somatic depolarization, transforms the cell’s response to basal input from one axonal spike to a 20 ms burst of three spikes, an output that is highly informative because it is rare in spontaneous activity. **(C)** Inferences concerning dendritic spikes in neocortical pyramidal neurons. Apical tufts and thus the AIZ of pyramidal neurons receive inputs from diverse sources of contextual information. Calcium currents, and thus synaptic plasticity, depend on backpropagating action potentials (bAPs, gray), apical dendritic calcium spikes (red) and NMDA spikes (blue). NMDA spikes require both local depolarization and glutamate (blue dots). **(A,B)** are from Figure 2 of Larkum (2013) (permission to reuse acknowledged) and **(C)** is modified from Figure 1 of Larkum and Phillips (2016) (courtesy of Cambridge University Press).

This section briefly outlines evidence that the apical dendrites that receive information from diverse sources, including feedback, are critical for context dependent gating of feedforward inputs via their effects on the AIZ. For more detailed reviews see (Larkum, 2013) and (Phillips, 2017). Depolarization of the AIZ, either antidromically or synaptically, can generate calcium dependent regenerative potentials in the apical dendrite, sometimes referred to as calcium spikes, though they are longer lasting than regular sodium spikes (Larkum and Zhu, 2002). Calcium spiking is greatly facilitated by back-propagating sodium action potentials in an interaction referred to as back-propagation activated calcium-spike firing (BAC-firing). BAC-firing can therefore turn a single axonal spike into a high frequency burst (Larkum et al., 2001; Larkum and Zhu, 2002; Williams and Stuart, 2002) as shown in Figure 1. This provides a cellular mechanism by which pyramidal cells can respond more strongly to their basal inputs when that is amplified by depolarizing synaptic input to their apical synapses in layer 1 (see reviews by Larkum, 2013; D’Souza and Burkhalter, 2017).

**Figure 2:**
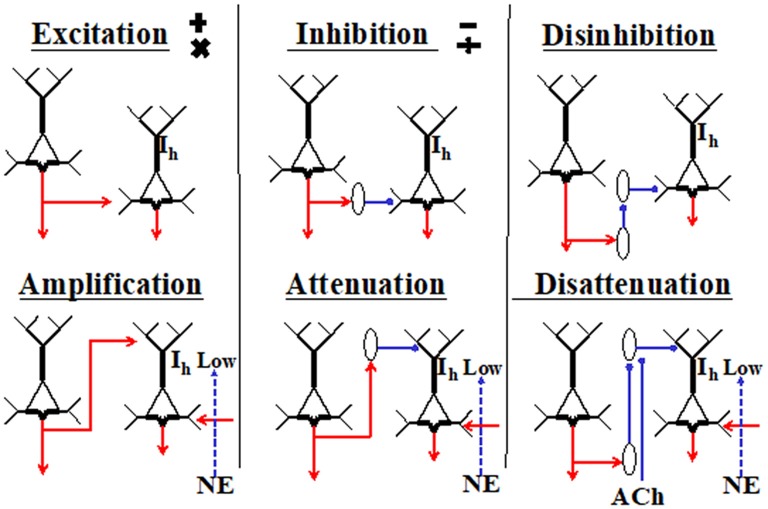
Primitive interactions from which neocortical circuits are built (adapted from Phillips et al., 2015, 2016). These generic depictions of neocortical pyramidal cells distinguish their apical and basal dendrites. The diagrams are not to be interpreted as microcircuits, and no attempt is made to show any columnar organization. The two cells shown in each section could be in either the same or different columns, or in different cortical regions. Six different ways in which the cell on the left could affect activity of the cell on the right are shown. Hyperpolarization-activated currents through HCN channels are shown as I_h_. They are crucial because when high, they disconnect the AIZ from the soma. The diagram is intended to indicate that the effects of apical input are more conditional than those of basal inputs because they require low I_h_ and the presence of net basal excitation. Inhibitory interneurons are shown as ovals. Cholinergic inputs are shown as ACh. The adrenergic inputs that reduce I_h_ are shown as norepinephrine (NE). There are many ways in which these primitives could be combined. For example, the outputs of a given pyramidal cell could be excitatory at some of its projective sites and amplifying at others, or an inhibitory interneuron could combine disinhibition with disattenuation by inhibiting interneurons that target the soma as well as those that target the tuft.

Excitatory inputs to apical synapses in layer 1 are complemented by inputs from inhibitory interneurons residing within that layer, as well as from interneurons in lower layers that project their axons into layer 1, such as the somatostatin-expressing Martinotti cells (reviewed by Gentet et al., 2012; Jiang et al., 2015). Because the effects of synaptic inputs to the AIZ depend upon spiking initiated by basal input, these effects have been interpreted as mechanisms for amplifying or attenuating response to the basal input, as shown in Figure 2 (Phillips et al., 2016). Anatomical evidence also suggests some such interpretation because it shows that inputs to apical dendrites come from a diverse range of sources, including feedback from higher cortical regions, whereas input to basal dendrites come from a restricted range of sources such as those that specify receptive field selectivity. As feedback has often been associated with modulatory functions in perceptual systems (e.g., Zhang et al., 2014), this is in broad agreement with our interpretation of the intracellular evidence as indicating that apical inputs can function in a mode in which they amplify the cell’s responses to basal inputs when relevant and attenuate them when irrelevant.

There are several reasons for assuming that much feedback from higher cortical regions is not driving, including the simple observation that if it were then cells lower in the hierarchy of neocortical abstractions would inherit the large receptive fields of those projecting to them from higher in the hierarchy, but this is rarely observed. There are many ways in which inputs could influence a cell’s output without being driving, however, and several of these ways could plausibly be referred to as being modulatory even though their information processing functions differ greatly from each other (Kay et al., 2017; Kay and Phillips, 2018). The information processing functions of the AIZ and the diverse inputs that it receives are therefore not adequately described by classifying them as “modulatory”. For present purposes suffice it to say that the kind of contextual modulation likely to be implemented by the AIZ is that required to interpret ambiguous stimuli in the most probable way, where the notion of “ambiguity” is interpreted broadly so as to include ambiguity of presence or task relevance, as well as ambiguity of categorization. Consider A 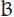 C, for example. The context used to disambiguate the central symbol does so even though that context is neither necessary nor sufficient to see the central symbol. The input to be disambiguated is both necessary and sufficient in that even in the absence of context the ambiguous input is seen if and only if there is something to be seen. The ambiguity of the central symbol in this particular demonstration can be brought to awareness by noting that the central symbol in 12 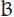 14 is likely to be interpreted differently, though it is identical to that shown in a different context. This phenomenological demonstration of contextual modulation can be related to apical function by assuming that in the context of other letters activity of the neural population code interpreting the ambiguous symbol as a letter is amplified, and that interpreting it as a digit is attenuated. If on first seeing the ambiguous symbol in the context of letters the reader was aware of the possibility of it being a number rather than a letter, then we assume that that awareness had less salience or clarity than its interpretation as a letter. Recent advances in information theory have now been used to show that this kind of contextual modulation is very different from multiplicative and divisive operations as well as from subtraction, none of which are plausibly implemented by BAC-firing (Kay et al., 2017; Kay and Phillips, 2018).

Most of the research on BAC-firing has been on layer 5 cells, but pyramidal cells in both infragranular and supragranular layers have apical dendrites in layer 1, and, there is evidence that something similar also occurs in other cells, such as those in Layer 2/3 of rat somatosensory (Palmer et al., 2014) and prefrontal cortex (Boudewijns et al., 2013). These findings suggest that intracellular mechanisms for the contextual amplification of relevant signals may be widely distributed throughout the cortex in both infra- and supra-granular pyramidal cells.

Anatomical evidence clearly indicates that apical inputs to neocortical pyramidal cells come from a wide variety of sources. These include direct feedback from higher cortical regions, indirect feedback via the thalamus, long-range lateral connections both within and between cortical regions, and the amygdala (Gur and Snodderly, 2008; Rubio-Garrido et al., 2009). This wide range contrasts greatly with the narrow range of sources specifying their selective sensitivity, which predominantly target basal and perisomatic sites. Though some inputs to layer 1 do connect to inhibitory interneurons, their synapses are predominantly depolarizing (Shao and Burkhalter, 1996) and on the apical tufts of pyramidal cells (Budd, 1998). This is all as expected on the assumption that apical inputs are predominantly amplifying, rather than driving.

The enhancement of axonal output by apical input is referred to as AA (e.g., Bachmann, 2015; Phillips et al., 2015; Takahashi et al., 2016; Phillips, 2017). Reduction of this effect is referred to as disamplification, or attenuation, which differs from other forms of inhibition or suppression because, even when strong, it does not prevent output, but simply reduces amplification of that output. Evidence for AA comes from many sources including both *in vitro* and *in vivo* cellular physiology (Larkum, 2013; Phillips et al., 2016; Takahashi et al., 2016; Phillips, 2017) and from macroscopic neuroimaging (Muckli et al., 2015; Petro and Muckli, 2017).

As indicated in Figure 2, the effects of apical inputs on axonal spiking are highly dependent on the state of the HCN-channels that pass hyperpolarization-activated currents (I_h_) (for a review see Biel et al., 2009). They have a high density in the apical tufts of L5 neocortical pyramidal neurons (e.g., Lörincz et al., 2002). These non-synaptic cation conductances are tonically active at rest and act as a leak conductance that tends to isolate the apical inputs from the soma unless I_h_ is low. As Figure 2 shows, we hypothesize that the extent to which apical inputs amplify or attenuate response to basal inputs is regulated by the adrenergic system via its effects on I_h_. This central hypothesis has now been directly confirmed by research using two-photon dendritic Ca^2+^ imaging and *in vivo* whole-cell and extracellular recordings in awake mice (Labarrera et al., 2018). As their research is independent of ours, the extent to which it provides empirical support for our hypotheses greatly increases our confidence in their validity and importance. Thus, adrenergic arousal tends to increase the extent to which apical inputs influence axonal output, with crucial implications for apical function and its potential relevance to the state of consciousness, as discussed in further detail in the next section.

## Evidence and Arguments Relating Apical Function to Conscious State

There are a wide variety of grounds on which apical function can be related closely to conscious state (e.g., Bachmann, 2015; Phillips et al., 2016), each of which is briefly outlined here. We do not claim that these grounds are conclusive, either separately or collectively. Our aim here is to show that apical function can be associated with conscious state on several different grounds. Though not conclusive, these grounds are extensive, and involve anatomy, physiology, psychophysiology, psychophysics, macroscopic neuroimaging, computational modelling, pathology and philosophy.

The anatomical grounds are in part that apical synapses are a major target for the long-range connections that have often been associated with consciousness. Apical inputs are diverse and include feedback from regions higher in the neocortical pathway as well as inputs from higher-order thalamus (for reviews see: Gur and Snodderly, 2008; Rubio-Garrido et al., 2009; D’Souza and Burkhalter, 2017). Perceptual awareness of particular stimuli is thought to be dependent on interactions between feedforward and feedback signals on several grounds (van Gaal and Lamme, 2012; Pinto et al., 2013). First, conscious processing is associated with greatly increased recurrent long-range interaction (e.g., Gaillard et al., 2009; Koch et al., 2016). Second, recurrent interactions between primary and higher visual areas are correlated with the ability to report the presence or absence of a stimulus in both humans and other animals (e.g., Super et al., 2001; Haynes et al., 2005). Using a multidisciplinary approach, Manita et al. (2015) demonstrate that an interaction between somatosensory and recurrent motor signals is essential for accurate perception in mice. In accord with our hypotheses, apical synapses played a leading role in this interaction. Third, psychophysical studies show that masking prevents conscious perception of a target by interfering with recurrent interactions and/or by directing facilitation to the masking stimulus instead of to the target stimulus (e.g., Bachmann, 1994, 1997; Lamme et al., 2002; Del Cul et al., 2007; Koivisto and Revonsuo, 2010). This evidence suggests that, even when awake and attending, backward-masked stimuli can fail to reach consciousness because the relevant feedforward signals are masked before the recurrent feedback has time to affect processing at the lower levels (e.g., Bachmann, 1994, 1997, 2012; Lamme and Roelfsema, 2000; Lamme, 2004; Bachmann and Hudetz, 2014). Fourth, the sequence from normal consciousness to the minimally conscious state, the vegetative state, and coma is strongly associated with decreasing amounts of recurrent interaction (e.g., Bekinschtein et al., 2009; Vanhaudenhuyse et al., 2010; see however Tzovara et al., 2015). Fifth, transcranial magnetic stimulation (TMS) indicates that both the content and the level of consciousness depend on these recurrent interactions and the associated cortical effective connectivity and integration (e.g., Pascual-Leone and Walsh, 2001; Jolij and Lamme, 2005; Massimini et al., 2005; Murphy et al., 2016).

Apical function has been directly related to behavioral evidence of perceptual awareness by Takahashi et al. (2016) They found that, as predicted by the hypothesis of AA, overt detection of small whisker deflections by awake mice is closely correlated with calcium currents in the superficial layers. Great technical difficulties are involved in directly assessing apical function in awake behaving animals, however, so such direct evidence for an association between apical function and perceptual awareness is as yet rare.

A more plentiful source of evidence relating apical function to conscious state concerns the effects of adrenergic arousal, because that plays a leading role in regulating conscious state, and affects apical function partly via its effects on I_h_ (see Phillips et al., 2016 for an in-depth review). In brief, when adrenergic levels and arousal are minimized, as in slow wave sleep for example, consciousness is absent or minimal, and apical contributions to pyramidal cell spiking are also minimal because high levels of the leak current I_h_ tend to isolate apical sites from the soma. Thus, in such states, consciousness and amplification of selected signals are both minimal. Adrenergic input is stronger when awake, however, and this enables the amplification of selected signals partly because it enhances communication between apical sites and the soma. Adrenergic arousal affects apical sites in particular because norepinephrine varicosities and HCN channels are both most dense in the superficial cortical layers, and NE tends to reduce I_h_ (e.g., Audet et al., 1988; Lörincz et al., 2002; Wang et al., 2007; Agster et al., 2013). Furthermore, in the awake state, transient and sustained changes in arousal, attention, and locomotion are highly correlated with noradrenergic and cholinergic activity (McGinley et al., 2015). Thus, the degree of alertness and the extent to which attention is focused involves rapid modifications of neocortical activities by the noradrenergic and cholinergic systems, which modify I_h_ and K^+^ currents in the apical dendrites of selected cells (Harnett et al., 2013, 2015). It is well established that adrenergic arousal increases the effects of prioritization on conscious perception, memory and learning (Mather et al., 2016). The research reviewed by Mather et al. (2016) and discussed by peers shows that prioritized stimuli and their neighbors in space and time, “grab attention”, with major consequences for conscious cognition and action. AA provides an intracellular mechanism by which such prioritization can be achieved (Larkum and Phillips, 2016; Phillips et al., 2016). As emotionally salient events are particularly likely to be prioritized (Mather et al., 2016) this may help explain why the amygdala projects directly to apical synapses (Amaral et al., 2003).

Higher-order thalamus, which provides much of the input to apical synapses in layer 1, is often associated with perceptual awareness, attention, and working memory. Many studies show that its various regions regulate the strength with which signals are transmitted between neocortical regions, but without corrupting their information content (reviewed in Sherman, 2007, 2012, 2016; Nakajima and Halassa, 2017). This has been most often shown in primary sensory regions of rodents, but it has also been shown in PFC (e.g., Schmitt et al., 2017); and in primates (e.g., Purushotaman et al., 2012; Komura et al., 2013; Marion et al., 2013; Zhou et al., 2016). The crucial contributions of higher-order thalamus to cortical information processing have been studied in many ways. For example, Zhou et al. (2016) found that pulvinar deactivation in rhesus monkeys caused severe deficits in attentive modulation of and behavioral responding to neocortical signals from the affected parts of the visual field. This was combined with a localized increase in low-frequency oscillations associated with inattention or sleep, suggesting that interactions of each part of visual cortex with an associated region in ventro-lateral pulvinar are required to maintain that part of cortex in an active state of attentive awareness. Komura et al. (2013) showed that in monkeys higher-order thalamic activity is closely related to the animal’s confidence when making perceptual decisions. A perceptual categorization task was used to evaluate perceptually experienced content, and an opt-out task was used to explore the subjects’ confidence levels. They found that LGN activity was correlated with the content of the behavioral decision, whereas pulvinar activity was correlated with the monkey’s confidence in that decision. All these findings support the fundamental distinctions between signal strength and information content in the neural domain, and between levels of consciousness and semantic content in the subjective domain. They clearly indicate that, though certain versions of the distinction between level and content may be questionable (Bayne et al., 2016), some such distinction is needed.

Apical function is implicated in the functions of higher-order thalamus because it sends major projections to layer 1. It is possible that the higher-order thalamic afferents to cortex that are used to amplify those local cortical activities that are currently relevant project to layer 1, whereas those that become part of what the cell’s output transmits information about project to lower layers. A simple but clear hint concerning the distal dendritic locations of amplifying feedback signals is provided by the LGN where descending afferents from layer 6 cells of V1 project to the distal dendrites of the thalamic relay cells (Sherman, 2012, 2016). In accordance with this theme of distal locations for amplifying inputs, Rubio-Garrido et al. (2009) show that a large number of higher-order thalamic afferents converge on pyramidal cell distal dendrites in layer 1 throughout sensory, association, and motor regions of rat neocortex. Some of these higher-order thalamic afferents branch to innervate large parts of the neocortex, whereas others arborize within a single region (Collins et al., 2018; Halassa, 2018). Wimmer et al. (2010) found that in rats the posteromedial nucleus of the thalamus projects densely and specifically to layers 1 and 5A of vibrissal somatosensory cortex. Roth et al. (2016) find that in mice a higher-order thalamic nucleus conveys diverse contextual information to sensory cortex via layer 1. Another body of evidence implicating apical synapses in the transmission of amplifying higher-order thalamic information to cortex is provided by studies of the bush baby, a small nocturnal primate. Purushotaman et al. (2012) show that activating neurons in the lateral pulvinar of bush babies can strongly boost the responses of cells in corresponding regions of V1 to their direct input from LGN while suppressing responses to surrounding regions. They also found that reversible inactivation of the lateral pulvinar cells prevented V1 cells transmitting their visual information beyond V1, such that the animals gave no behavioral signs of being aware of the suppressed stimuli. These higher-order thalamic effects on signal strength are likely to be mediated predominantly via apical synapses because Marion et al. (2013) found that in bush babies the lateral pulvinar projects densely to layer 1 of V1 but to no other layers in that region.

Functional neuroimaging and perturbational methods for assessing the complexity of cortical activity provide further evidence that long-range interactions, which typically send large projections to apical synapses, are crucial to the dynamics of conscious states (e.g., Långsjö et al., 2012; MacDonald et al., 2015; Casarotto et al., 2016). High-resolution 7-Tesla fMRI also provides supportive evidence by showing that in awake humans the superficial layers of early sensory cortex receive diverse contextual inputs (e.g., Muckli et al., 2015; Petro and Muckli, 2017; Petro et al., 2017).

Computational modeling shows explicitly that, as we have assumed, noradrenergic input to the neocortex from the locus coeruleus can selectively enhance or attenuate cortical responses (Safaai et al., 2015; Todd and Manaligod, 2018). Furthermore, there have been several studies of computational capabilities that arise from neurons with an apical compartment that can either amplify or attenuate response to basal inputs. Reviews of these computational studies show that all of the capabilities modeled either require consciousness or elevate selected activities into consciousness (Phillips et al., 2015; Phillips, 2017).

Several psychopathologies provide further grounds for relating apical function to disorders of consciousness. Impaired apical function may be involved in psychotic reductions in context-sensitivity (Phillips and Silverstein, 2003, 2013). This will be discussed in more detail below in relation to schizophrenia and the effects of ketamine on NMDA receptors. Though there are likely to be several other relevant disorders, there is space here to mention only one other example. It has been shown that a rapid decline in expression of HCN1 channels and I_h_ precedes the onset of seizures in a genetic rat model of absence epilepsy (Kole et al., 2007). This loss of HCN1 occurred mainly in the apical dendrites of layer 5 pyramidal neurons in the cortex. Dual whole-cell recordings by Kole et al showed that this increased somato-dendritic coupling and significantly reduced the threshold for generation of dendritic calcium spikes by backpropagating action potentials. Thus, this provides a somato-dendritic mechanism for increasing the general non-selective AA and synchronization of cortical output, which could play an important role in the generation of absence seizures. Absence seizures due to a general increase in this somato-dendritic coupling is yet further evidence that it is not AA *per se* that is associated with conscious states, but selective amplification.

Finally in this list of grounds for relating apical function to conscious state, we note that some philosophers have argued that apical function could provide a mechanism by which semantic contents can become conscious given that they have been computed by other processes (Marvan and Polák, 2017). This is of importance because it implies that, contrary to a common philosophical assumption, production of the semantic contents of a phenomenal quality and it’s becoming conscious are not one and the same thing. From the perspective of the dual model of consciousness advocated by Marvan and Polák (2017), production of phenomenal qualities can be associated with the feedforward processing that is mediated predominantly by basal dendrites, whereas consciousness is associated with the apical influences. As this is assumed to occur within a recurrent hierarchy of abstractions, changes to the salience of signals at any one hierarchical level will have major implications for the processing of feedforward signals at others, and thus for consciously experienced semantic content as a whole.

Putative relations between conscious state, adrenergic arousal, I_h_, and apical function that we infer from these findings and arguments are summarized in Table 1.

**Table 1:** A summary of hypothesized relations between brain state, norepinephrine levels (NE), HCN currents (I_h_), and apical dendritic function.

State	NE level	I_h_	Apical function
Asleep	Low	High	Isolated from soma
Awake	Moderate	Moderate	Selective amplification
Attentive	High	Low	Inc. Selectivity & Amplif.
High arousal	Maximum	Minimum	Selective drive e.g., Stereotypical impulses	
Anesthetized Ketamine propofol Isoflurane	Low	Minimum	Non-selective slow waves

*Though the distinction between consciousness and unconsciousness may be regarded as categorical, we assume that the distinction between different waking and attentive states is neither categorical nor mutually exclusive, being reflected in variations of both the level of arousal and the semantic contents of consciousness. A moderate level of selective amplification is hypothesized to provide the field of consciousness when awake, with amplification being greater at the focus of attention. Many other differences between the states listed, such as those in cholinergic and other neuromodulatory systems, are not shown. Evidence that selectivity of prioritization increases with arousal level is reviewed and assessed by peers in Mather et al. (2016) (“Inc.” refers to “increase.” “Stereotypical impulses” are those leading to reflexive, habitual, or impulsive responses unconstrained by their appropriateness to current circumstances, and which become more probable when stress is high. “Non-selective slow waves” include the synchronised activity of very many cells such as those often observed during slow-wave sleep or anesthesia)*.

Table 1 shows that apical depolarization may be amplifying under some conditions and driving under others. There it is assumed that if HCN channels are fully closed such that I_h_ is at a minimum then apical inputs can become part of the drive that pyramidal cells receive. This has been shown directly by Atkinson and Williams (2009) using HCN antagonists combined with apical current injection. We hypothesize that this can occur when humans are stressed because behavior reverts to reflexive habitual responding unconstrained by its appropriateness to current circumstances when high levels of glucocorticoid stress hormones and adrenergic arousal are combined (Schwabe et al., 2012; reviewed in Arnsten, 2015). Other evidence that apical inputs can be driving under special circumstances comes from the studies relating apical function to the detection of small deflections of a whisker by mice (Takahashi et al., 2016). They found high false alarm rates in some conditions, which implies that responses usually dependent upon the actual occurrence of a deflection also sometimes occurred when there was no deflection. This suggests that, at some point in the neural pathway leading to behavior, context that normally serves to facilitate detection of an actual deflection of the whisker can become driving and produce a “detection” response by itself. Table 1 implies that this is more likely to happen under conditions of high arousal, and that this is in part mediated by adrenergic suppression of I_h_. All these inferences are testable, thus opening the door to many theoretically motivated empirical investigations. For example, the studies of Atkinson and Williams (2009) were performed *in vitro*, so our assumption that their findings are relevant needs to be tested. Another inference to be tested concerns false alarms, such as those observed by Takahashi et al. (2016). If, as hypothesized in Table 1, false alarms are due to an I_h_-dependent increase in apical drive when stress is high, then false alarms should increase with stress in a way that is reduced by adrenergic antagonists. Finally, it must be tempting to investigate whether the proneness to hallucinatory experiences produced by inter-stimulus conditioning (Powers et al., 2017) is also related to AA or to effects on the strength of driving feedforward synaptic connections.

## Evidence That General Anesthetics Interfere With Apical Function

Apical function can be related to general anesthesia on several grounds. Anesthetic-induced unconsciousness occurs at doses lower than that required to block feedforward transmission to sensory cortex, and is associated with selective loss of modulatory feedback (e.g., Lamme et al., 1998; Alkire et al., 2008), which is predominantly mediated by apical synapses. Evidence that general anesthetics can reduce dendritic spine density within tens of minutes (Colon et al., 2017) also suggests a link to apical synapses.

**Figure 3:**
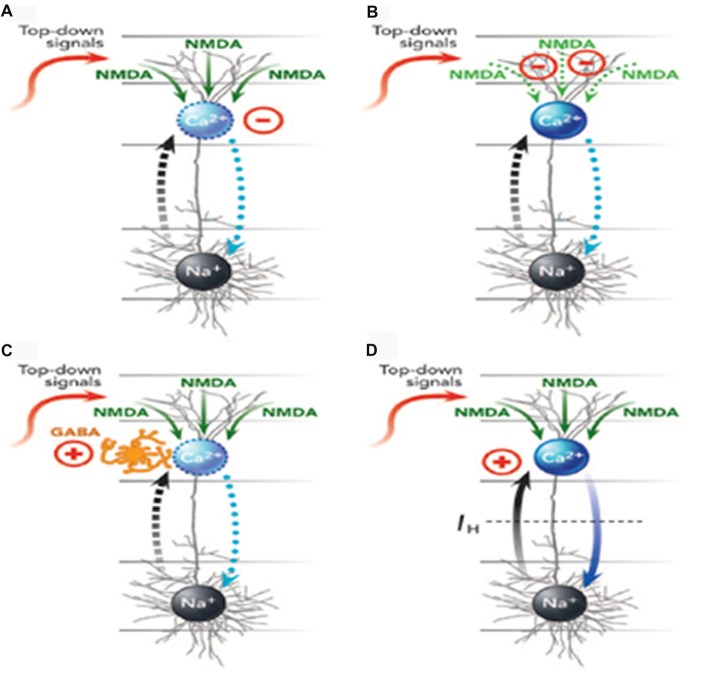
Four ways **(A–D)** in which anesthetics can interfere with apical function in neocortical pyramidal cells (from Meyer, 2015, with permission from Elsevier). The blue oval is the AIZ. The black oval is the somatic integration zone that generates action potentials. See the main text for further explanation and examples of anesthetics that affect each of the four mechanisms. GABA, γ-aminobutyric acid; NMDA, N-methyl-d-aspartate; Na^+^, sodium channels; Ca^2+^, calcium channels.

Mechanisms by which different anesthetics can interfere with apical function in different ways are reviewed by Meyer (2015) and depicted in Figure 3. Here we simply summarize his conclusions.

Meyer (2015) distinguished four ways in which general anesthetics could interfere with apical function, with many of them interfering in more than one way. First, they could suppress the generation of calcium action potentials by the AIZ (Figure 3A). Anesthetics identified as having this effect include isoflurane, urethane, and pentobarbital. Second, they could suppress regenerative N-methyl-d-aspartate (NMDA) potentials that carry signals from peripheral tuft dendrites toward the AIZ (Figure 3B). Anesthetics identified as having this effect include ketamine and nitrous oxide. Third, anesthetics can up-regulate inhibitory interneurons that, in turn, suppress the generation of dendritic calcium action potentials (Figure 3C). Anesthetics identified as having this effect include propofol and barbiturates. Fourth, anesthetics can block the hyperpolarization-activated current I_h_, which is the leak conductance that uncouples somatic and dendritic compartments under normal conditions. When I_h_ is blocked, somatic activation alone may trigger a dendritic calcium spike and thus a burst of somatic action potentials, leading to a breakdown of the mechanism by which only a few selected outputs are amplified while many others are suppressed (Figure 3D). Anesthetics identified as having this effect include ketamine, isoflurane, propofol, halothane and pentobarbital.

On the basis of this evidence Meyer (2015) concluded that higher-order thalamocortical projections and corticocortical top-down projections have key roles in conscious perception, and share two properties: (1) their activity is decreased under general anesthesia; and (2) they both terminate primarily in the superficial neocortical layers that contain apical dendrites. Meyer (2015) interpreted evidence for a close association between general anesthesia and apical function in terms of predictive coding theory. The evidence for close associations between consciousness, anesthesia and apical function is clear by itself, however, and does not depend upon the validity of any form of predictive coding theory. In particular, we do not see anything in the evidence that either he or we have reviewed that implies that feedforward driving signals code for the difference between feedforward and feedback signals, i.e., “prediction errors”.

Thus, bottom-up pathways from subcortical neuromodulators that regulate levels of general arousal and top-down pathways that amplify specific contents could both do so via effects on apical function. Anesthetics that Mashour and Hudetz (2017) describe as primarily affecting the level of consciousness, such as dexmedetomidine, could in part do so by affecting the inputs to apical dendrites from subcortical mechanisms that regulate the general level of arousal. Anesthetics that Mashour and Hudetz (2017) describe as primarily affecting the contents of consciousness, such as ketamine, could do so predominantly by impairing selective amplification of relevant signals by thalamocortical inputs to apical dendrites. If only a limited subset of neurons selectively signaling some sensory features is active at the subthreshold level, then widespread (non-selective) modulation arriving at the apical dendrites can effectively single-out only that small subset (Bachmann, 1994, 2014; Larkum and Phillips, 2016; Phillips et al., 2016). Thus, the effects of general subcortical arousal and local intracortical amplification are interdependent. The intracortical interactions specify what to prioritize and subcortical arousal regulates the extent to which prioritized signals are amplified (Mather et al., 2016). Therefore it is likely that all or most anesthetics will to some extent affect both the level of consciousness and its semantic contents.

Put simply, these observations suggest that to be conscious is to have particular explicitly reportable percepts, thoughts, feelings and intentions, and that general anesthetics affect this by interfering with the intracellular processes by which particular activities are selectively amplified when relevant to the current context.

## How Can Both Increases and Decreases of I_H_ Lead to a Loss of Consciousness?

Many fascinating issues arise concerning relations between anesthesia, adrenergic arousal, slow-wave sleep, and REM sleep. We do not have space to comment on all of them here, but we do discuss one issue that may puzzle some readers. This concerns the apparent contradiction between findings indicating that consciousness is lost both when I_h_ is high, as in sleep, and when it is blocked by ketamine, propofol, and isoflurane (Chen et al., 2005, 2009; Zhou et al., 2013). This apparent contradiction can be resolved in various ways. For example, one plausible explanation is that the opening of background “leak” potassium channels by general anesthetics may suppress any apical contributions to action potential generation in spite of a parallel reduction in I_h_. Another possibility is that by so strongly suppressing I_h_ in all pyramidal neurons, anesthetics may reduce the extent to which particular neuronal responses can be selectively amplified. This is in accord with findings indicating that heterogeneity of single-cell responses within larger ensembles provides a more accurate neural correlate of detection and/or perception than the overall mean level of response (Tononi et al., 2016; Storm et al., 2017). It is also in accord with the non-selective loss of I_h_ through HCN1 channels that is associated with absence seizures in a rat model of epilepsy (Kole et al., 2007). Finally, this emphasis upon the increased heterogeneity of signals strengths due to selective amplification is in agreement with evidence that general anesthetics enhance global synchronization similar to that observed in slow-wave sleep, thus reducing the differentiation and complexity of cortico-thalamic activity (Tononi et al., 2016).

## The Atypical Effects of Ketamine Provide Further Evidence That States of Consciousness Depend on Apical Function

Mashour and Hudetz (2017) emphasize the atypical effects of ketamine. In contrast to other general anesthetics it suppresses ventrolateral preoptic nucleus (VLPO), does not depend upon increasing GABAergic inhibition, activates arousal-promoting nuclei, depends on norepinephrine for its hypnotic action, increases cortical acetylcholine levels, and preserves electrophysiological and other signs of wakefulness (Mashour, 2014). Nevertheless, despite some preserved functions, the contents of the dissociated state of consciousness induced by sub-anesthetic doses of ketamine are disorganized, disconnected, and incoherent (Collier, 1972). Instead of predominantly reflecting the environment, the bizarre imagery experienced in response to ketamine has internal origins. Overall, this fits well with evidence that ketamine affects neural activity and conscious content via its effects on NMDA receptors (Phillips and Silverstein, 2003, 2013). This also fits well with apical function because AA is highly dependent upon the regenerative, voltage-dependent activation of NMDA receptor channels in the form of NMDA-spikes (Larkum et al., 2009; Palmer et al., 2014). Furthermore, although ketamine has effects that contrast with those of other anesthetics, it, like them, has large effects on apical spiking both *in vitro* and *in vivo* (Potez and Larkum, 2008).

One advantage of this perspective on NMDA function is that it suggests how psychotic experiences in schizophrenia can arise from reduced current through NMDA receptor channels. The close association between psychosis and reduced NMDA currents is well established (Phillips and Silverstein, 2003; Moghaddam and Javitt, 2012), but it has long been difficult to explain how that could have such specific effects given that NMDA and AMPA receptors are usually co-localized. This puzzle can now be resolved by noting that apical contributions to action potential generation require NMDA spikes, whereas basal contributions do not (Palmer et al., 2014). It has been found that ketamine impairs the ability to form coherent percepts (e.g., Uhlhaas et al., 2007), as does schizophrenia (Phillips and Silverstein, 2003). Given that apical input has modulatory functions, we can now propose an explanation of how it is that at sub-anesthetic doses ketamine selectively impairs the ability to use context to form coherent thoughts and percepts. First, note that neural activity can be driven by internal knowledge and thoughts, as well as by external input. If that internal drive operates via basal dendrites then input from other parts of the system to the apical tuft could function to ensure that the internally generated activity as a whole is coherent. If the effects of input to the apical tuft are impaired by a ketamine-induced reduction of NMDA currents then memories and thoughts will be less coherent, as hypothesized by Phillips and Silverstein (2003), and being less amplified will be less likely to be transmitted to other cortical regions.

## These Issues May Be Clarified by Recent Advances in Information Theory

A well-known conceptual framework that has been used to address the issue of the neural bases of variations in conscious state and relate them to anesthesia is that of integrated information theory (IIT) (e.g., Hill and Tononi, 2005; Alkire et al., 2008; Oizumi et al., 2014; Sarasso et al., 2015; Tononi et al., 2016). Though that theory makes no explicit reference to subcellular mechanisms such as apical function, a similar theory does, i.e., the theory of coherent infomax (Phillips et al., 1995, 2015; Kay and Phillips, 2011; Kay et al., 1998; Phillips et al., 2016). From the IIT perspective, anesthetics have been described as hyperpolarizing neurons by increasing inhibition, increasing intrinsic potassium conductance, and/or by decreasing excitation (Alkire et al., 2008). The coherent infomax perspective implies that, in addition to any such effects, anesthetics may also operate by selectively increasing attenuation and/or decreasing amplification via intracellular and microcircuit mechanisms such as those reviewed above.

Information theory and the distinction between “modulatory” and “driving” interactions are central to both IIT and the theory of coherent infomax. It has until recently been difficult to provide a rigorous formalization of the notion of modulation, however, because that requires an adequate analysis of multivariate mutual information. Although it will come as a surprise to many, mutual information in classical Shannon information theory was formulated only for the case of a single input vector and a single output vector. Multivariate mutual information is not well defined in that theory. Shannon tried to generalize the theory to encompass multi-variate mutual information, but did not succeed. This situation is changing rapidly, however, and several ways of partitioning multivariate mutual information have now been proposed (e.g., Williams and Beer, 2010; Harder et al., 2013; Bertschinger et al., 2014; Griffith and Koch, 2014; Ince, 2017), and a Special Issue of the journal Entropy (2018) has been devoted to them (Information Decomposition of Target Effects from Multi-Source Interactions). These advances have been used to provide rigorous conceptual and analytic tools for specifying neural goal functions (Wibral et al., 2017), and for distinguishing between modulatory and driving interactions (Kay et al., 2017; Kay and Phillips, 2018). These conceptual and analytic tools have not yet been used to study general anesthesia, but the hypotheses outlined here predict that when they are, they will provide further evidence that conscious state and anesthesia involve selective contextual modulation operating via apical dendrites.

## Unresolved Issues and Difficulties

Several testable predictions have been noted above, but other many unresolved issues and difficulties arise. BAC-firing, AA, and the effects of adrenergic arousal, as outlined above, have so far been observed only in mammalian neocortex. Thus, if consciousness in general were to be identified with system-level properties that require such structures and processes it may well turn out to have the implausible implication that only mammals are conscious. Furthermore, though the weight of evidence and opinion gives neocortex a central role in the NCC in humans, it has been argued that, even in humans, consciousness is possible without a neocortex (Merker, 2007). Merker (2013) argues that even in the presence of a neocortex the neuronal basis of phenomenological sensory content may be more accurately located in higher-order thalamic regions, such as the dorsal pulvinar, than in neocortex. In contrast to that, we have assumed that in humans it is located in thalamocortical activities as a whole. It is not yet clear whether and how such issues can be resolved. One hypothetical possibility is that higher order thalamus acts as a substrate for “primitive consciousness”, with its contents also being “primitive”. Another difficulty is that the HCN channels that we have hypothesized to mediate the effects of adrenergic arousal on apical function and mental state are absent in neonates and develop slowly over a long time-span (Atkinson and Williams, 2009). Our hypotheses therefore seem to imply developmental changes of mental state that are so fundamental that many may find them disconcerting, as to some extent do we. One way to deal with difficulties that seem to implausibly restrict the range of states considered to be conscious may be to put more emphasis upon the diversity of conscious states rather that on what they all have in common, if anything. This raises many untested predictions and unresolved issues. Can the use of context-sensitive abilities to selectively amplify or strengthen relevant activities be implemented in ways other than that in mammalian neocortex, and if so how do constraints on those various ways differ? Do uniquely human mental states arise from the evolution of enhanced intracellular capabilities for dynamically deciding what to amplify? If so, to what extent does that explain cognitive capabilities unique to humans?

Difficulties and unresolved issues are clearly raised by considering dreams from the perspective of the hypotheses outlined in this article. Table 1 shows that apical inputs are isolated from the soma by high I_h_ when levels of noradrenaline are low, as they are during sleep. Noradrenaline levels are low during REM sleep (Hobson et al., 1975; Aston-Jones and Bloom, 1981; Rasmussen et al., 1986; Takahashi et al., 2010), however; so, if conscious state depends upon apical function, this seems to suggest that dreams may not be conscious while being dreamt, but only become conscious when remembered on awaking. Some people affirm that to be consistent with their own phenomenology, and Marvan and Polák (2017) could classify dreams as an example of content without consciousness in support of their dual coding theory. That theory contradicts the common identification of consciousness with content that is phenomenally experienced, however, and many people believe that they are conscious of their dreams while having them. These issues concerning consciousness may not be well posed, however. As there are clear differences between all three states on what grounds can dream states be grouped with waking rather than with slow-wave sleep? Consider dreams during anesthesia (e.g., Brandner et al., 1997; Eer et al., 2009). A fruitful way forward may therefore be to focus on clarifying the fundamental similarities and differences between various mental states while leaving open the question as to whether they can all be adequately categorized as being either conscious or non-conscious. Here the concept of levels of consciousness may again be useful because the phenomenology of dreamt content is often vague, faint, fragmented, unstable and lacking in deliberate control by the dreamer.

Many other issues concerning the dependence of anesthetic effects on subject and mental state arise. How can the effects of anesthetics emphasized above be distinguished from their effects on the cholinergic, dopaminergic, and seretonergic systems? Are their effects on non-mammalian species compatible with the hypotheses proposed above? Do their effects change with development from infancy as would be expected given that some of the effects of some of the anesthetics are mediated by HCN channels that are not present at birth and develop slowly? How are the effects of ketamine on I_h_, and thus on apical function, related to the transition from subanesthetic to anesthetic doses? Are subanesthetic dissociative states induced by ketamine explicable as effects on the contents rather than on the level of consciousness? Are there uniquely human effects of ketamine, as suggested by its psychotomimetic effects and the doubts that many psychiatrists and others have concerning the validity of animal models of psychoses? If anesthesia is interpreted as blocking the amplifying effects of context that we have related to consciousness, then how can they be observed in animals under anesthesia, as they have been (e.g., Purushotaman et al., 2012; Palmer et al., 2014)? Though the discoveries of Labarrera et al. (2018) indicate that the regulation of AA via the effect of norepinephrine on I_h_ can be observed under isoflurane anesthesia as well as during waking it is not yet clear to us why the anesthesia did not suppress those effects.

Finally, many questions arise concerning the distinction between driving and modulatory interactions, such as amplification and attenuation, and the relation of that to the distinction between feedforward and feedback signals. Much evidence for a distinction between driving and modulatory effects of glutamate at the cellular level has been provided by studies of Class 1 monosynaptic inputs, which have many properties expected of drivers, and Class 2 inputs, which many properties expected of modulators (Sherman, 2007, 2012, 2016). In the above we have assumed that feedforward signals are driving and that feedback signals are modulatory, as do many others. The evidence reviewed by Sherman clearly contradicts that assumption, however, because categorizing synaptic connections as Class 1 or 2 clearly indicates that feedforward modulation and feedback drive are also common (e.g., Covic and Sherman, 2011; De Pasquale and Sherman, 2011). Several fundamental questions arise. What is the relation between the class of synaptic interactions and the morphological site of the synapses? This is as yet unknown. An obvious default assumption is that Class 1 is predominantly basal and Class 2 is predominantly apical, but it is not known whether that is so or not. Although we doubt that things are so simple, it may be that the descending connections to deeper layers provide driving connections via basal dendrites while those in layer 1 provide modulatory influences via apical synapses. Do driving feedback connections that descend the neocortical hierarchy serve short-term memory and the creative imagination? Does driving feedback project to the same cells as those transmitting feedforward drive, and, if so, how can the cells to which they project disambiguate the signals that they receive? Does the prevalence of descending Class 1 driving connections support the counter-stream theory that already has anatomical and computational support (Ullman, 1995)? Could the presence of descending drive in the absence of adequate contextual modulation explain why dreams and delusions are so often incoherent?

Many more doubts and questions concerning these issues and hypotheses could be raised. It is not the aim of this article to dispel the doubts or to answer all the questions; it is to show that they merit far wider consideration.

## Author Contributions

WP initiated the article and wrote the first draft. WP, JS and TB developed the article.

## Conflict of Interest Statement

The authors declare that the research was conducted in the absence of any commercial or financial relationships that could be construed as a potential conflict of interest.
